# Connecting lignin-degradation pathway with pre-treatment inhibitor sensitivity of *Cupriavidus necator*

**DOI:** 10.3389/fmicb.2014.00247

**Published:** 2014-05-27

**Authors:** Wei Wang, Shihui Yang, Glendon B. Hunsinger, Philip T. Pienkos, David K. Johnson

**Affiliations:** ^1^National Renewable Energy Laboratory, Biosciences CenterGolden, CO, USA; ^2^National Renewable Energy Laboratory, National Bioenergy CenterGolden, CO, USA

**Keywords:** *Cupriavidus necator*, pre-treatment inhibitor, saccharified slurry, deacetylation, lignin degradation, biofuel, polyhydroxylbutyrate (PHB), genomics

## Abstract

To produce lignocellulosic biofuels economically, the complete release of monomers from the plant cell wall components, cellulose, hemicellulose, and lignin, through pre-treatment and hydrolysis (both enzymatic and chemical), and the efficient utilization of these monomers as carbon sources, is crucial. In addition, the identification and development of robust microbial biofuel production strains that can tolerate the toxic compounds generated during pre-treatment and hydrolysis is also essential. In this work, *Cupriavidus necator* was selected due to its capabilities for utilizing lignin monomers and producing polyhydroxylbutyrate (PHB), a bioplastic as well as an advanced biofuel intermediate. We characterized the growth kinetics of *C. necator* in pre-treated corn stover slurry as well as individually in the pre-sence of 11 potentially toxic compounds in the saccharified slurry. We found that *C. necator* was sensitive to the saccharified slurry produced from dilute acid pre-treated corn stover. Five out of 11 compounds within the slurry were characterized as toxic to *C. necator*, namely ammonium acetate, furfural, hydroxymethylfurfural (HMF), benzoic acid, and p-coumaric acid. Aldehydes (e.g., furfural and HMF) were more toxic than the acetate and the lignin degradation products benzoic acid and p-coumaric acid; furfural was identified as the most toxic compound. Although toxic to *C. necator* at high concentration, ammonium acetate, benzoic acid, and p-coumaric acid could be utilized by *C. necator* with a stimulating effect on *C. necator* growth. Consequently, the lignin degradation pathway of *C. necator* was reconstructed based on genomic information and literature. The efficient conversion of intermediate catechol to downstream products of cis,cis-muconate or 2-hydroxymuconate-6-semialdehyde may help improve the robustness of *C. necator* to benzoic acid and p-coumaric acid as well as improve PHB productivity.

## Introduction

Lignocellulosic biomass is considered a renewable and sustainable source for energy production. The great environmental, energy security, and economic benefits of biofuels have driven research on biomass throughout the world, resulting in several pilot and demonstration scale projects, based primarily on cellulosic ethanol. One of the leading routes for cellulosic ethanol production is based on the deconstruction of biomass to a monomeric sugar solution produced by chemical pre-treatment and enzymatic saccharification termed a saccharified slurry (hereafter referred to as slurry) followed by fermentation of the sugars to ethanol. However, the physico-chemical properties of ethanol limit its further penetration into the current petroleum-based transportation fuel infrastructure (Serrano-Ruiz and Dumesic, 2011). The lower energy density of ethanol due to its higher oxygen content compared to conventional petroleum hydrocarbons, is a significant disadvantage to using bioethanol as a fuel alternative.

Current transportation fuel infrastructure has been established for hydrocarbon-based petroleum. Many advanced biofuels under development exhibit advantages such as higher energy densities, longer carbon chains and lower oxygen numbers. For example, there are some fuels derived from fatty acids with carbon numbers in the range from C_12_ to C_22_ that can be upgraded to high-energy-density hydrocarbons via hydrotreating. Therefore, development of hydrocarbon fuels, which are compatible with the current fuel infrastructure as replacements or blendstocks for gasoline, jet, and diesel, has recently received great attention.

The primary routes leading to hydrocarbon synthesis from biomass are thermochemical, biochemical, and hybrid approaches which employ elements of both. Bacteria, yeasts, or fungi can naturally synthesize fatty acids, isoprenoids, and polyalkanoates for energy storage. Although these compounds are mostly exploited in pharmaceutical, nutritional, and packaging sectors, they also have great potential for production of hydrocarbon fuels (Zhang et al., 2011). However, several obstacles need to be solved for future hydrocarbon production at the commercial scale. Firstly, although compounds, such as acetate, furfural, and HMF that are produced during chemical pre-treatments (e.g., dilute acid pre-treatment) and have been investigated extensively due to their toxicity to ethanologens (Olsson and HahnHagerdal, 1996; Liu et al., 2004, 2005, 2008; Gorsich et al., 2006; Endo et al., 2008; Franden et al., 2009, 2013; Allen et al., 2010; Bowman et al., 2010; Yang et al., 2010a,b, 2012b; He et al., 2012; Ask et al., 2013; Bajwa et al., 2013; Wilson et al., 2013), there are few reports on the effect of these compounds on hydrocarbon producers (Huang et al., 2012). Various mechanisms and approaches have been proposed and applied for decreasing inhibition in cellulosic hydrolysates (Olsson and HahnHagerdal, 1996; Larsson et al., 1999; Zaldivar and Ingram, 1999; Zaldivar et al., 1999; Petersson et al., 2006; Endo et al., 2008; Franden et al., 2009, 2013; Mills et al., 2009; Yang et al., 2010a,b, 2012a,b; Ask et al., 2013; Bajwa et al., 2013; Iwaki et al., 2013), but strategies for strain improvement have yet to be applied (Dunlop, 2011; Dunlop et al., 2011; Kang and Chang, 2012).

Moreover, the efficient utilization of lignin, a major cell component, would greatly improve the economics of advanced biofuel production. Due to its highly recalcitrant aromatic structure, lignin is seen largely as an impediment to biochemical processes due to its reinforcement of the cellulose/hemicellulose matrix complicating the saccharification process (Zeng et al., 2014). Owing to the high energy state of its aromatic ring structures, lignin could have great value as a substrate for energy production, but so far very limited opportunities for microbial transformation of lignin to high value-added fuels or fuel intermediates have been identified. Some bacteria are known to degrade lignin monomers via the β-ketoadipate pathway (Harwood and Parales, 1996). Currently lignin is almost untouched in lignocellulosic ethanol fermentations and remains behind in the fermentation residue after distillation. These residues are mostly burned to provide power in current cellulosic ethanol production processes, which places a low value on this material. To reach the goal of utilizing lignin as a carbon source for advanced biofuel production, the composition of lignin degradation compounds as well as the toxic effects of these compounds on hydrocarbon-producing microorganisms should be investigated.

In this study we are seeking a biological understanding of the impact of model inhibitors, which include lignin degradation products as well as furans and acetate, on hydrocarbon-producing microorganisms. *Cupriavidus necator* (Syn. *Alcaligenes eutrophus, Ralstonia eutropha*) has been extensively studied for production of polyhydroxybutyrate (PHB), which consists of a C_4_ repeating unit that can be thermally depolymerized and then decarboxylated to propene (Fischer et al., 2011; Pilath et al., 2013), an intermediate, which can be upgraded to hydrocarbon fuels via commercial oligomerization technologies. *C. necator* has been reported to be able to utilize lignin monomers as a carbon source (Pérez-Pantoja et al., 2008).

The genome sequence of *C. necator* H16 has been published with *in silico* genome modeling and a developed genetics system (Pohlmann et al., 2006; Park et al., 2011; Brigham et al., 2012). In addition, several transcriptomic studies have recently been reported (Peplinski et al., 2010; Brigham et al., 2012), and the genome sequences for a number of other *Cupriavidus* spp. are also now available (Amadou et al., 2008; Pérez-Pantoja et al., 2008; Janssen et al., 2010; Lykidis et al., 2010; Poehlein et al., 2011; Cserhati et al., 2012; Hong et al., 2012; Van Houdt et al., 2012; Li et al., 2013). This information will facilitate future comparative genomics and systems biology studies to develop *C. necator* H16 as a robust and metabolically diverse hydrocarbon-intermediate production strain. Genomics is applied in this study to explore the metabolic pathways related to lignin utilization and response to toxic compounds in slurries, which will provide perspectives for strain metabolic engineering toward future economic hydrocarbon production using lignin.

## Materials and methods

### Strains and media

The strain used in this study is a glucose-utilizing mutant of *C. necator* H16 (wild-type H16 is not able to metabolize glucose) (Orita et al., 2012), *C. necator* 11599, which was purchased from NCIMB culture collection. It is routinely cultured in LB at 37°C. A minimal medium recipe was selected for this study (Cavalheiro et al., 2009). Specifically, the defined minimal medium for *C. necator* (per liter, pH 6.8) was: 10 g glucose, 1.0 g (NH_4_)_2_SO_4_, 1.5 g KH_2_PO_4_, 9 g Na_2_HPO_4_ 12H_2_O, 0.2 g MgSO_4_ 7H_2_O, 1.0 mL trace element solution. The Trace Element Solution (per liter): 10 g FeSO_4_.7H_2_O, 2.25 g ZnSO_4_.7H_2_O, 0.5 g MnSO_4_.5H_2_O, 2 g CaCl_2_.2H_2_O, and 1 g CuSO_4_.5H_2_O, 0.23 g Na_2_B_4_O_7_.10H_2_O, 0.1 g (NH_4_)_6_M_O7_O_24_, 10 mL 35% HC1.

### Production of saccharified slurry and mock media

A deacetylated saccharified slurry, which was produced from the modified sulfuric acid pre-treatment and enzymatic hydrolysis of corn stover including an added deacetylation step before pre-treatment, was used in this study (Chen et al., 2012). The composition of the mock sugar media simulating the saccharified slurry is summarized in Table 1. The composition is based on the composition of the saccharified slurry in fermentation media at the level of 20% total solids.

**Table 1:** **Composition of mock saccharified hydrolysate slurry**.

	**Glucose (g/L)**	**Xylose (g/L)**	**Acetate (mM)**
Mock slurry (1X)	100.4	51.4	36.6

### Growth of *C. necator* on saccharified slurry

*Cupriavidus necator* was first grown in 5 mL of LB in 125 mL baffled flasks, cultured at 200 rpm, and 37°C. After 1 day, a 10% inoculum was added to 50 mL of fermentation media in a 250 mL flask and incubated in a shaker at 37°C and 180 rpm for 4 days. The fermentation media contained either mock sugar slurry as shown in Table 1 or saccharified slurry supplemented with tryptone (10 g/L) and yeast extract (5 g/L) as nutrients. Mock slurry was added at a level to achieve the same sugar concentrations (e.g., the glucose concentration in the 2X-diluted mock medium was 50 g/L). All experiments were run in duplicate.

### PHB analysis

The PHB content of the bacterial cells was determined by a quantitative method that used HPLC analysis to measure the crotonic acid formed by acid-catalyzed depolymerization of PHB (Karr et al., 1983). Cell mass samples were freeze-dried before analysis. PHB-containing dried bacterial cells (15–50 mg) were then digested in 96% H_2_SO_4_ (1 mL) at 90°C for 1 h. The reaction vials were then cooled on ice, after which, ice-cold 0.01N H_2_SO_4_ (4 mL) was added followed by rapid mixing. The samples were further diluted 20- to 150-fold with 0.01N H_2_SO_4_ before analysis by HPLC.

The concentration of crotonic acid was measured at 210 nm using an HPLC equipped with a photodiode array detector (Agilent 1100, Agilent Technologies, Palo Alto, CA). A Rezex RFQ Fast Acids column (100 × 7.8 mm, 8 μm particle size, Phenomenex, Torrance, CA) and Cation H+ guard column (BioRad Laboratories, CA) operated at 85°C were used to separate the crotonic acid present in the reaction solutions. The eluent was 0.01N H_2_SO_4_ at a flow rate of 1.0 mL min^−1^. Samples and crotonic acid standards were filtered through 0.45 μm pore size nylon membrane syringe filters (Pall Corp., NY) prior to injection onto the column. The HPLC was controlled and data were analyzed using Agilent ChemStation software (Rev.B.03.02).

### Characterization of potential inhibitors

The selection of chemicals for sensitivity assays was based on analyses of saccharified slurry by GC-MS and LC-MS analyses performed at NREL as well as ICP-MS analyses carried out by Huffman Labs (Golden, CO). The compounds identified with high concentrations in the slurry and their potential derivatives were selected for further investigation. The compounds included ammonium (added to neutralize pre-treated corn stover) with two common anions of acetate (released by hydrolysis of hemicelluloses) and sulfate (from the sulfuric acid pre-treatment); sugar degradation products furfural and HMF; lignin monomers vanillin, coumaric acid, ferulic acid, and 4-hydroxybenzaldehyde as well as benzoic acid, a common intermediate from lignin monomer (coumarate and cinnamate) degradation (Figure 5). We also included products from the oxidation of lignin monomers, vanillic acid and 4-hydroxybenzoic acid, in a similar concentration range to their aldehyde forms (Table 2). The 1X concentrations for some chemicals (e.g., benzoic acid) in the slurry used for testing are based on concentrations used in a previous study.

**Table 2:** **The chemicals and their concentration ranges (mM) selected for toxicity testing**.

**Concentration Range tested (Fold)**	**0.1×**	**0.25×**	**0.5×**	**1×**	**2.5×**	**5×**	**10×**
**AMMONIUM SALTS**							
Ammonium Acetate (AA, 36.6 mM)	4.64	11.6	23.2	46.4	116.1	232.2	464.4
Ammonium Sulfate (AS, 21mM)	4.0	10.0	20.0	40.0	100.0	200.0	400.0
**FURANS**							
Furfural (F, 12.3 mM)	1.26	3.15	6.30	12.59	31.48	62.97	125.94
HMF (1.3 mM)	0.14	0.36	0.71	1.43	3.57	7.14	14.27
**LIGNIN MONOMERS**							
4-Hydroxybenzaldehyde (HBA, 0.08 mM)	0.01	0.02	0.04	0.08	0.20	0.41	0.82
Vanillin (V, 0.13 mM)	0.01	0.03	0.07	0.13	0.33	0.66	1.31
Benzoic Acid (B, 0.037 mM)	0.08	0.20	0.41	0.82	2.05	4.09	8.19
p-Coumaric Acid (CA, 0.73 mM)	0.07	0.18	0.37	0.73	1.83	3.65	7.31
Ferulic Acid (FA, 0.46 mM)	0.05	0.12	0.23	0.46	1.16	2.32	4.63
4-Hydroxybenzoic Acid (HB, 0.20 mM)	0.01	0.04	0.07	0.14	0.36	0.72	1.45
Vanillic Acid (VA, 0.054 mM)	0.01	0.03	0.06	0.12	0.30	0.59	1.19

The concentration (mM) following the chemical abbreviation is the concentration in the slurry calculated from analytical results of GC-MS, LC-MS, or ICP-MS.

### Bioscreen C high throughput toxicity assay

The high-throughput Bioscreen C assay was carried out as reported previously (Franden et al., 2009, 2013). Briefly, *C. necator* cells were revived from overnight LB culture with OD_600nm_ adjusted to 3.0 using minimal medium. This cell suspension was used as seed culture to inoculate Bioscreen C plates containing 290 μ L minimal medium per well at an initial OD_600nm_ of 0.1. Growth was then monitored using the Bioscreen C instrument (GrowthCurves USA, NJ) with three technical replicates. The experiments were repeated at least two times.

*Cupriavidus necator* grown in the absence of potential inhibitory compounds was used as the control, and inhibition studies utilized cultures of *C. necator* challenged with different concentrations of each compound ranging from 0.1- to 10-fold (0.1X to 10X, Table 2). Stock solutions of compounds at 10X concentrations were prepared by dissolving the compounds to be tested in the minimal medium. These stock solutions were then diluted in minimal medium for testing at lower concentrations. For certain compounds with low aqueous solubility, incubation at 55–60°C in a hot dry bath for several hours was needed for complete dissolution. The pH of the stock solutions was adjusted to the desired point of 6.8 using ammonium hydroxide (NH_4_OH) or sulfuric acid (H_2_SO_4_) and then filter sterilized before using.

### Genomic investigation for lignin degradation pathway reconstruction

To identify the enzymes related to lignin degradation, the protein sequences of *C. necator* H16 were extracted and reannotated functionally. Briefly, 6626 protein sequences were downloaded from NCBI (Genbank#: AM260479) and imported into CLC Genomics Workbench (V5.5) as the reference protein sequences for Blast search. In addition, the protein sequences were also imported into Blast2GO for the functional annotation (Gotz et al., 2008). The Kyoto Encyclopedia of Genes and Genomes (KEGG) pathways were then extracted as well as the information of euKaryotic Orthologous Groups (KOG), enzyme code, and the reaction substrate(s) and product(s). The potential homologous gene(s) in *C. necator* H16 were identified by re-iterated BlastP searches. The information on protein product and conserved domains were examined and the pathway was reconstructed with the enzyme and pathway information from a literature search.

#### Gas chromatographic analysis

Analysis of samples was performed on an Agilent 7890 GC equipped with a 5975 MS (Agilent Technologies, Palo Alto, CA). Sample compounds were separated using a 30 m × 0.25 mm × 0.25 mm DB-FFAP column (Agilent). HP MSD Chemstation software (Agilent) equipped with NIST database Rev. D.03.00 was used to determine the identity of the unknown compounds found within the samples.

Each sample was placed on an auto-sampler (Agilent) and injected at a volume of 1 μ L into the GC-MS (Agilent). The GC-MS method consisted of a front inlet temperature of 250°C, MS transfer line temperature of 280°C, and a scan range from 35 to 550 m/z. A starting temperature of 50°C was held for 1.5 min and then ramped at 11°C/min to a temperature of 165°C with no hold time, then continued at a ramped rate of 35°C/min to 250°C and held for 9.617 min. The method resulted in a run time of 28 min for each sample.

## Results and discussions

### Growth kinetics of *C. necator* in the saccharified slurry

**Figure 1:**
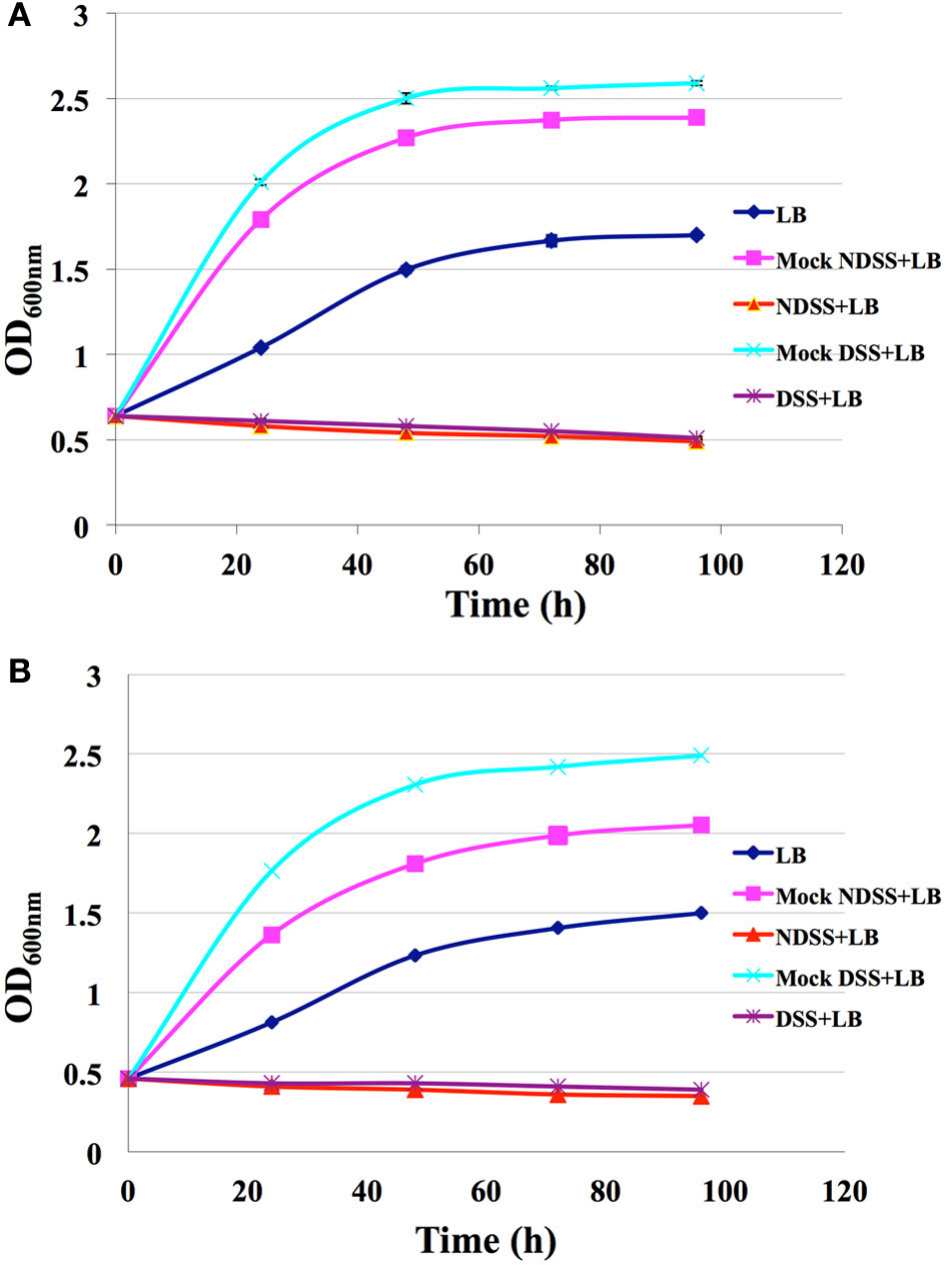
**Growth of *C. necator* in LB, mock media and pre-treated saccharified lignocellulosic slurry at original 1-fold concentration (A); or at a 2-fold dilution (B)**.

*Cupriavidus necator* did not grow in the original deacetylated saccharified slurry (Figure 1A) even in the 2-fold diluted saccharified slurry (Figure 1B) though it was able to grow in media containing the mock slurry. The sugars provided by the mock slurry allowed *C. necator* for superior growth over the LB alone in both 1-fold concentration and 2-fold dilution conditions with fast growth and high final OD_600nm_ value. This complete inhibition of growth in real slury suggests that *C. necator* is not tolerant to the combination of all toxic compounds present in the saccharified slurry.

### Kinetics of *C. necator* in the saccharified slurry after activated carbon (AC) treatment

It is well-known that acid pre-treatment of corn stover can generate various kinds of potential toxic inhibitors including organic acids and aldehydes, and can also add to the toxicity through the build up of inorganic salts from the sulfuric acid used for pre-treatment and ammonium hydroxide added for neutralization (e.g., organic and inorganic acids of levulinic acid, vanillic acid, hydroxybenzoic acid, sulfuric acid, and aldehydes of HMF and furfural). Furfural is generally considered the most potent inhibitor to various microbial catalysts such as *E. coli, Z. mobilis*, and yeast (Zaldivar et al., 1999; Liu et al., 2004, 2005, 2008; Gorsich et al., 2006; Allen et al., 2010; Bowman et al., 2010; He et al., 2012; Huang et al., 2012; Franden et al., 2013). The maximum amount of furfural was 2 g/L in the slurries in this study. Shake flask growth experiments showed that furfural greatly inhibited cell viability when the concentration was above 2 g/L (data not shown).

A chemical treatment process using activated carbon (AC) was applied in this study in order to improve growth by removing potential inhibitors such as furfural from the slurry. AC was added to the slurry at 0.1 g/mL loading and incubated for 2 h at 130 rpm, 24°C. The compositions of saccharified slurry before and after AC-treatment indicated that all the furfural and about 30% of the acetate was removed by the AC treatment (Table 3).

**Table 3:** **The compositions of deacetylated saccharified slurry before and after activated carbon (AC) treatment**.

**Composition**	**Untreated slurry**	**AC-treated slurry**
Cellobiose (g/L)	17.4	9.3
Glucose (g/L)	100.4	95.1
Xylose (g/L)	51.4	48.3
Arabinose (g/L)	10.1	10.4
Glycerol (g/L)	0.8	0.7
Acetic acid (mM)	36.6	26.6
HMF (mM)	ND	ND
Furfural (mM)	12.5	ND

ND, Not detected; the detection limitation for furfural is 2.6 mM, HMF is 2.8 mM.

**Figure 2:**
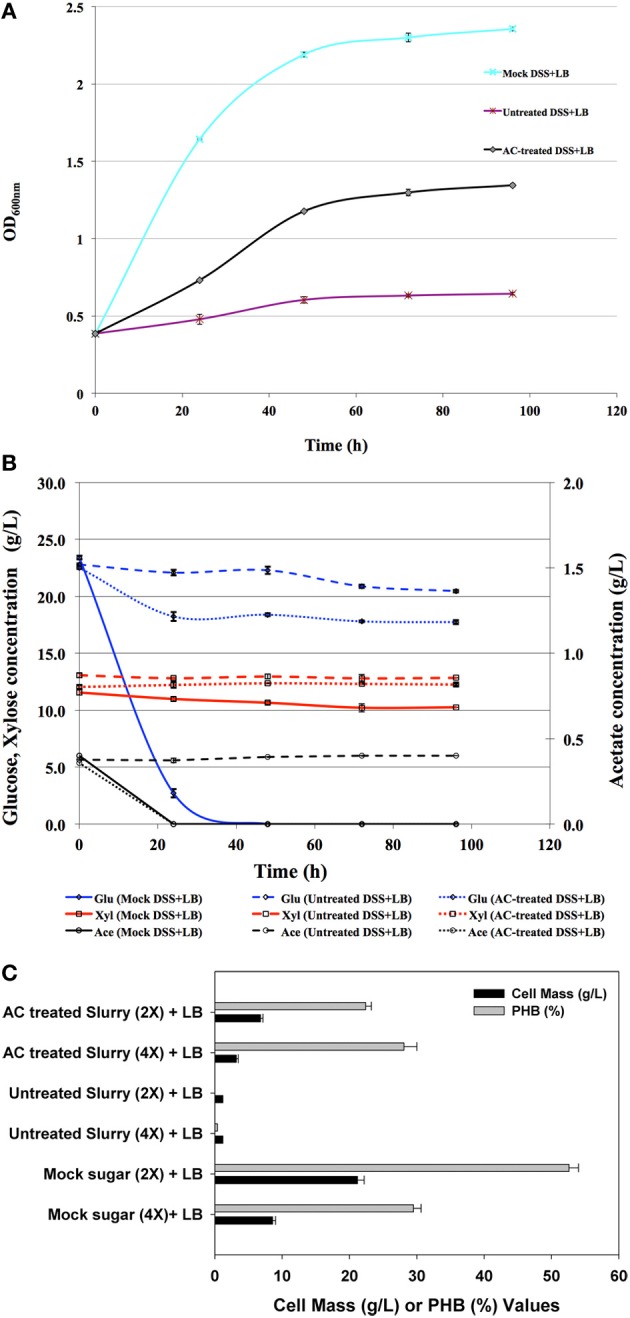
**Growth of *C. necator* on 4-fold (4×) diluted untreated and activated carbon (AC) treated saccharified slurry as well as the mock medium (A), and the corresponding sugar (Glu, glucose; Xyl, xylose) and acetate (Ace, acetate) concentrations (B), as well as cell mass and PHB yield with 2-fold or 4-fold dilution 96-h post-inoculation (C)**.

The impact of AC treatment on the toxicity of diluted saccharified slurry (4-fold dilution), which provided about 25 g/L of glucose to the growth media, was tested. As shown in Figure 2A, untreated saccharified slurry was very toxic to *C. necator*, while AC-treated saccharified slurry was much less inhibitory to growth, although it was still inhibitory compared to the mock slurry control. It is noteworthy that the consumption of glucose from AC-treated slurry was far slower than that from mock slurry and at 24 h, only about 25% of the total glucose was removed from the culture medium compared to 85% in the mock slurry (Figure 2B). This indicates that inhibitors remained in AC-treated slurry although furfural was completely removed. The remaining inhibitors are either unidentified residual components in the slurry (other than furfural) or components released from pre-treated corn stover during saccharification. It is noteworthy that besides the removal of all furfural by AC-treatment, our preliminary data indicated that a significant fraction of the other potentially toxic compounds such as acetic acid (Table 3) and lignin degradation products (e.g., vanillin, 4-hydroxybenzaldehyde, and p-coumaric acid) were also removed by AC-treatment. The impact of these individual toxic compounds in the saccharified slurry will be evaluated in the following section.

The cell yield from the AC-treated slurry (4-fold diluted, 4X) was about one third that of the culture grown on the mock slurry (4X), with similar amounts of PHB accumulated in the cells from both growth conditions 96 h post-inoculation (Figure 2C). However, when 2-fold diluted slurry was used, increased inhibition led to a lower cell mass and accordingly a lower PHB yield even with the carbon treated slurry (Figure 2C). All these results indicate that the toxicity of the biomass-derived slurry had a major impact on PHB production. Moreover, the low cell viability on 2x diluted slurry indicates that the inhibitory effect on cells is likely due to the combined contribution of several inhibitors besides furfural. Although the efficacy of the AC-treatment is obvious in term of cell growth, it only partially mitigated the inhibition. Identification of the other inhibitors in the AC-treated slurry is needed to help eliminate or mitigate the observed inhibition.

### Inhibitor sensitivity investigation using the high-throughput bioscreen C assay

**Figure 3:**
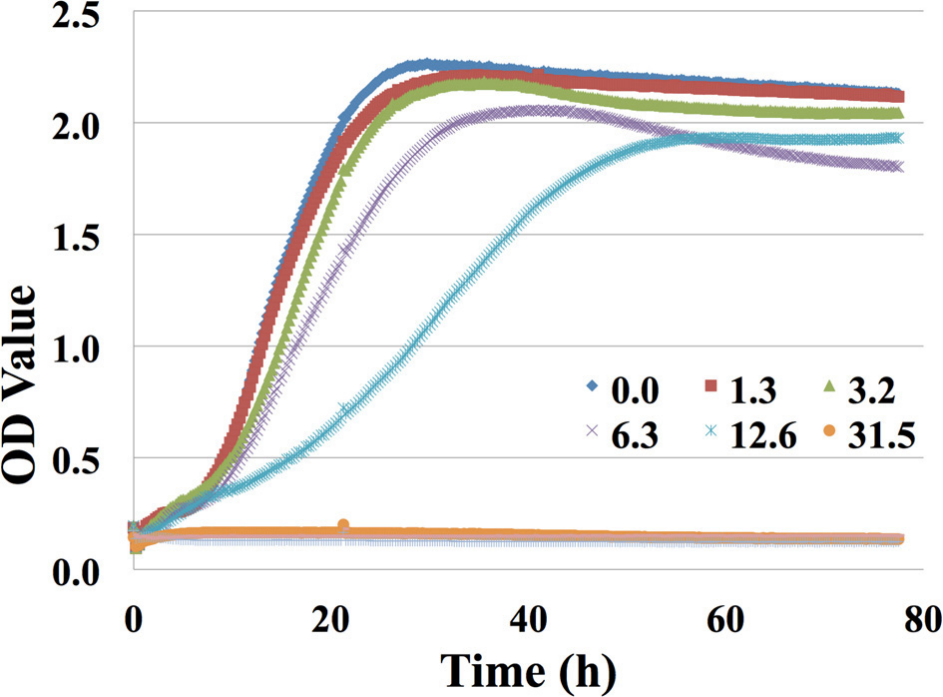
**Growth curves of *C. necator* in minimal medium with different concentrations (0, 1.3, 3.2, 6.3, 12.6, 31.5, 63, or 126 mM) of furfural using Bioscreen C assay**.

To systematically explore the impact of inhibitors in the saccharified slurry on *C. necator*, a high-throughput Bioscreen C growth assay was performed using growth medium augmented with the potentially toxic compounds that had been identified at concentration ranges discussed above (Table 2). Briefly, growth curves were generated by subtracting OD readings from the test wells in the Bioscreen C from the background wells containing blank medium only. Typical growth curves with *C. necator* grown with varied concentrations of furfural are shown in Figure 3. The high-throughput nature of this assay allowed for utilization of both biological replicates on multiple plates and technical replicates on the same plate. Generally speaking, correlation between growth curves in both technical and biological replicates was quite high (data not shown), emphasizing the power of this method. The results were consistent with previous shake flask experiments showing that furfural greatly inhibited cell growth when the concentration was above 2 g/L (ca. 20 mM).

Growth rates (μ in terms of h^−1^) for each growth curve were then calculated as described previously (Franden et al., 2009, 2013). The response values, given as the percentage of the growth rate compared with the control in the absence of supplemented inhibitor, were then calculated for each concentration (Table 4). The response values were then used to determine the concentration of inhibitor (IC50) that resulted in 50% growth compared to the control. The application of IC50 in this study was used to determine the top inhibitors in the slurries by comparing the IC50 of different compounds. This approach has frequently been used as a general toxicity indicator for potential inhibitors (Franden et al., 2013). IC50 values for 4-hydroxybenzaldehyde, vanillin, ferulic acid, 4-hydroxybenzoic acid, and vanillic acid were above the highest values tested, and the IC50 values for the remaining compounds were listed in Table 5. The growth of *C. necator* was inhibited by 5 out of the 11 compounds tested. The aldehydes, furfural and HMF, were the most toxic compounds in the slurry. The IC50 value of 9 mM for furfural was lower than the concentration detected in the saccharified slurry (12.6 mM or 1.2 g/L). Although the IC50 for benzoic acid had the lowest value, its concentration in the slurry was only 0.037 mM and less than 10% of the IC50 value making it the least toxic component in the slurry. The lignin degradation products were minimally toxic in the relevant concentration range except for p-coumaric acid with an IC50 value about 4 times higher than the concentration in the saccharified slurry. Ammonium acetate was found to be more toxic than ammonium sulfate (Table 5).

**Table 4:** **The growth rates and responses of *C. necator* to various furfural concentrations (mM) supplemented in the minimal medium**.

**Conc. (mM)**	**0**	**1.3**	**3.2**	**6.3**	**12.6**	**31.5**	**63**	**126**
Growth rate (μ)	0.19	0.18	0.13	0.12	0.06	0	0	0
Response (%)	100.0	97.5	66.6	61.1	32.1	0	0	0

**Table 5:** **The IC50 values and the ratio of the IC50 value to Conc. value (the concentration of the toxic compound identified in the saccharified slurry, mM) for *C. necator***.

	**Ammonium sulfate**	**Ammonium acetate**	**Furfural**	**HMF**	**Benzoic acid**	**p-Coumaric acid**
Conc. (mM)	21	36.6	12.6	1.43	0.037	0.73
IC50 (mM)	388	210	9	2.9	0.44	3.2
Ratio	18.5	5.7	0.7	2.0	11.9	4.4

A few key points, however, must be made that aren't captured in Table 4. One major observation is that some of the compounds found in saccharified slurry stimulate growth by as much as 50% (Table 6). Growth stimulation with some of these compounds (e.g., ammonium sulfate, ammonium acetate, benzoic acid, and coumaric acid) was seen at low concentrations, but these compounds became inhibitory at higher concentrations. This indicated that *C. necator* might utilize these compounds as supplemental carbon or nitrogen sources at low levels. The utilization of acetate by *C. necator* in shake flask experiments (data not shown) supports this hypothesis that the higher response values could be an indicator of growth stimulation by utilizing the supplemented substrates (Figure 2B), which is consistent with previous report that addition of acetate as a supplementary substrate improved the cell growth and PHB production in *C. necator* DSMZ 545 (Sharifzadeh Baei et al., 2009). The combination of growth stimulation by some compounds and inhibition by others complicated analysis of the impact of saccharified slurry on growth and productivities and should be further explored in the future.

**Table 6:** **Response values of *C. necator* to 11 potentially toxic compounds identified in the saccharified slurry at different concentrations (mM) supplemented in the minimal medium**.

**Conc. Range (Folds)**	**0×**	**0.1×**	**0.25×**	**0.5×**	**1×**	**2.5×**	**5×**	**10×**
Ammonium acetate	100	**112**	**154**	**145**	**115**	72	48	38
Ammonium sulfate	100	**122**	**122**	**114**	**107**	104	84	34
Furfural	100	98	67	61	32	0	0	0
4-Hydroxybenzaldehyde	100	100	100	77	93	105	91	108
HMF	100	87	76	73	59	48	43	31
Vanillin	100	97	98	101	98	92	68	58
Benzoic acid	100	**115**	**114**	**107**	96	60	38	29
Coumaric acid	100	101	**112**	**119**	**107**	79	46	26
Ferulic acid	100	108	99	99	101	103	101	89
4-Hydroxybenzoic acid	100	100	94	96	102	98	88	83
Vanillic acid	100	77	73	96	95	87	68	61

The values in bold font indicate stimulation of growth by the compound at the corresponding concentration.

**Figure 4:**
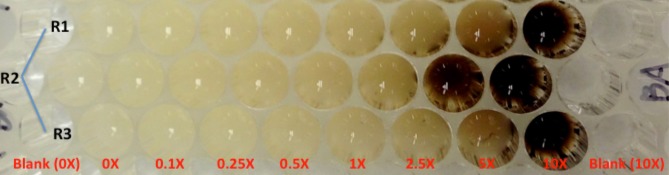
**The image of part of a Bioscreen C plate when culturing *C. necator* with minimal medium supplemented with benzoate at different concentrations, 5 days post inoculation**. R1, R2, and R3 are three technical replicate wells in the Bioscreen C honeycomb plate.

An additional point concerning the bacterial toxicity profiles is that they can provide additional information that could be used to understand microbial physiology and to propose genetic targets for metabolic engineering. For example, as the concentration of benzoic acid increased and the response changed from growth stimulation to inhibition, the color of the culture medium also became darker (Figure 4). This color change was not seen in un-inoculated control wells. The availability of the *C. necator* genome sequence will facilitate our understanding of this phenomenon using a genomics approach that will be explained in the following section.

### *C. necator* lignin-degradation pathway reconstruction

**Figure 5:**
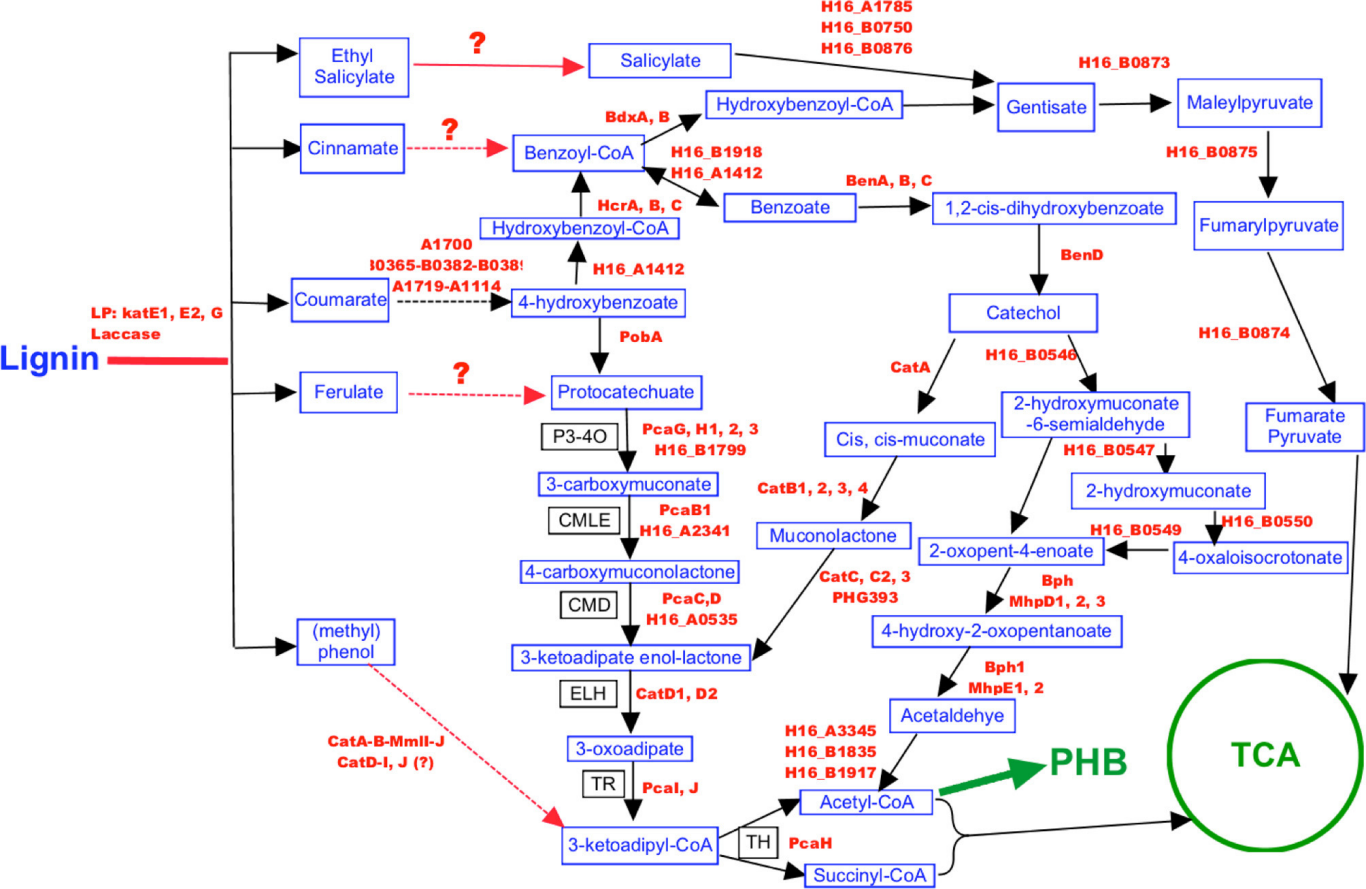
**Proposed lignin degradation pathway in *C. necator* H16**. Pathways and reactions with black solid lines have complete genes/enzymes identified in *C. necator* H16, while the red lines indicate those not completely identified. Genes encoding enzymes for metabolic reactions are bold red font.

*Cupriavidus necator* is capable of utilizing lignin monomers and recently a *Cupriavidus* sp. (*C. basilensis* B-8) has been reported to be able to utilize kraft lignin (Shi et al., 2013). With that information in hand, we then set out to reconstruct the lignin degradation pathway of *C. necator* based on genome information and literature reports to help understand the bottleneck for further development of *C. necator* as a lignocellulosic biofuel-production strain (Figure 5).

**Figure 6:**
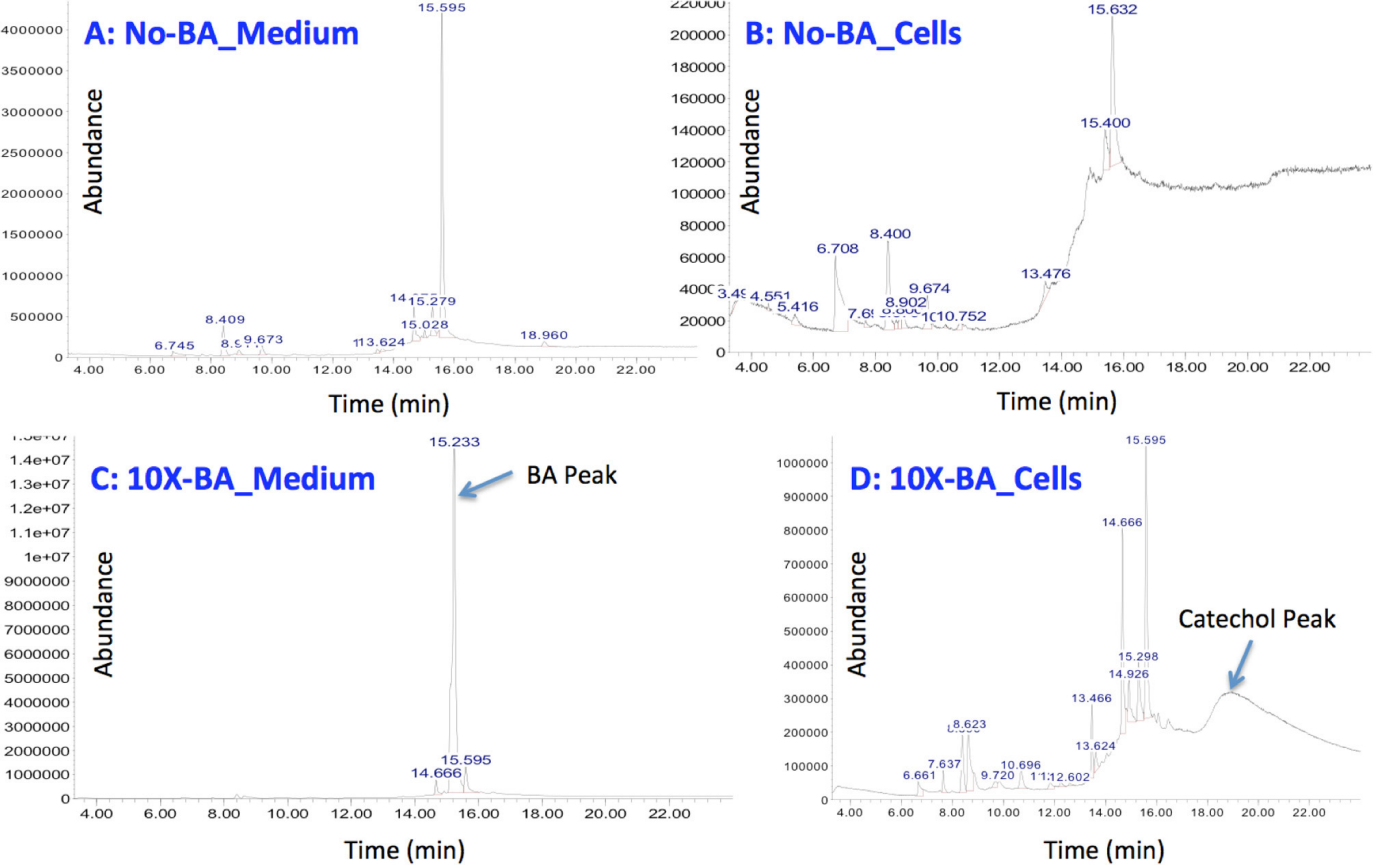
**GC-MS/MS result for supernatants from minimum medium only without benzoic acid supplementation (A), minimum medium only without benzoic acid supplementation but with *C. necator* inoculation (B), minimum medium with 10-fold (1 g/L) benzoic acid supplementation (C), minimum medium with 10-fold (1 g/L) benzoic acid supplementation, and *C. necator* inoculation (D)**.

Although the enzymatic reaction from lignin to lignin monomers and most reactions from lignin monomers to the metabolic intermediates are not yet fully revealed, homologous genes encoding the enzyme BenA, B, C, D were identified in *C. necator* that would convert benzoate to catechol. When the concentration of the supplemented benzoate is lower than the concentration characterized in the slurry, catechol will be further converted into cis,cis-muconate or 2-hydroxymuconate-6-semialdehyde through two different metabolic pathways leading to the accumulation of the key intermediate of acetyl-CoA for PHB production (Figure 5). Previous study already indicated that the accumulation of catechol in *Pseudomonas mendocina* can lead to its conversion of catechol into 1, 2-benzoquinone (Parulekar and Mavinkurve, 2006), which caused the culture medium to turn dark and at the same time inhibited the cellular growth since benzoquinone is a toxic agent against microorganisms such as *E. coli, Pseudomonas fluorescens*, and *Erwinia amylovora* (Beckman and Siedow, 1985). Our GC/MS study also indicated the correlation between the catechol appearance and benzoic acid disappearance when 1 g/L benzoic acid was supplemented into the minimum medium (Figure 6). Although further experimental data are needed to quantify the disappearance of benzoic acid and the appearance of catechol and 1,2-benzoqionone, our current study suggest that at high benzoate concentrations, *C. necator* can not convert catechol completely to cis,cis-muconate or 2-hydroxymuconate-6-semialdehyde and catechol began to accumulate which potentially caused the formation of 1, 2-benzoquinone and then turned medium into dark brown color and inhibited cell growth (Figures 4–6).

In addition, homologous genes were also identified in *C. necator* to carry out the reaction from p-coumarate to 4-hydrobenzoate and then feed into TCA cycle (Figure 5), which indicates that *C. necator* can utilize lignin monomer coumarate as carbon source for cell growth and may explain the stimulus effect of coumarate on *C. necator* growth in the lower concentration range. When high concentration of p-coumarate was supplemented into the medium, high concentration of catechol may be produced and the bottleneck reaction from catechol to cis,cis-muconate or 2-hydroxymuconate-6-semialdehyde (Figure 5) caused the inhibitory effect as discussed above. However, it is possible that other metabolic pathways that we have not been covered will also involved in and even play a key role on lignin monomer utilization and pre-treatment inhibitor sensitivity.

This study is an attempt and shows the feasibility to connect genomic information to explain microbial physiological phenomena. Although it could provide genetic targets for metabolic engineering to improve strain robustness (e.g., overexpression catechol dioxygenase to drive catechol into TCA instead of catechol accumulation), other experimental approaches such as systems biology study are needed to completely understand the mechanism of pre-treatment inhibitor sensitivity and genetic studies are especially required to confirm the hypothesis generated by bioinformatics study.

## Author contributions

Wei Wang, Shihui Yang, Philip T. Pienkos, and David K. Johnson designed the experiments. Wei Wang carried out the flask assay and AC treatment. Shihui Yang carried out the Bioscreen C assay and genomic study. Glendon B. Hunsinger performed the GC/MS. Shihui Yang and Wei Wang did the spectrum scanning. Wei Wang, Shihui Yang, Philip T. Pienkos, and David K. Johnson analyzed the data, and wrote the manuscript.

### Conflict of interest statement

The authors declare that the research was conducted in the absence of any commercial or financial relationships that could be construed as a potential conflict of interest.
