# Supplementary figures and images for: Connecting lignin-degradation pathway with pre-treatment inhibitor sensitivity of Cupriavidus necator
Source: Front Microbiol. 2014 May 27;5:247. doi: 10.3389/fmicb.2014.00247 (PMC4034039; doi:10.3389/fmicb.2014.00247)

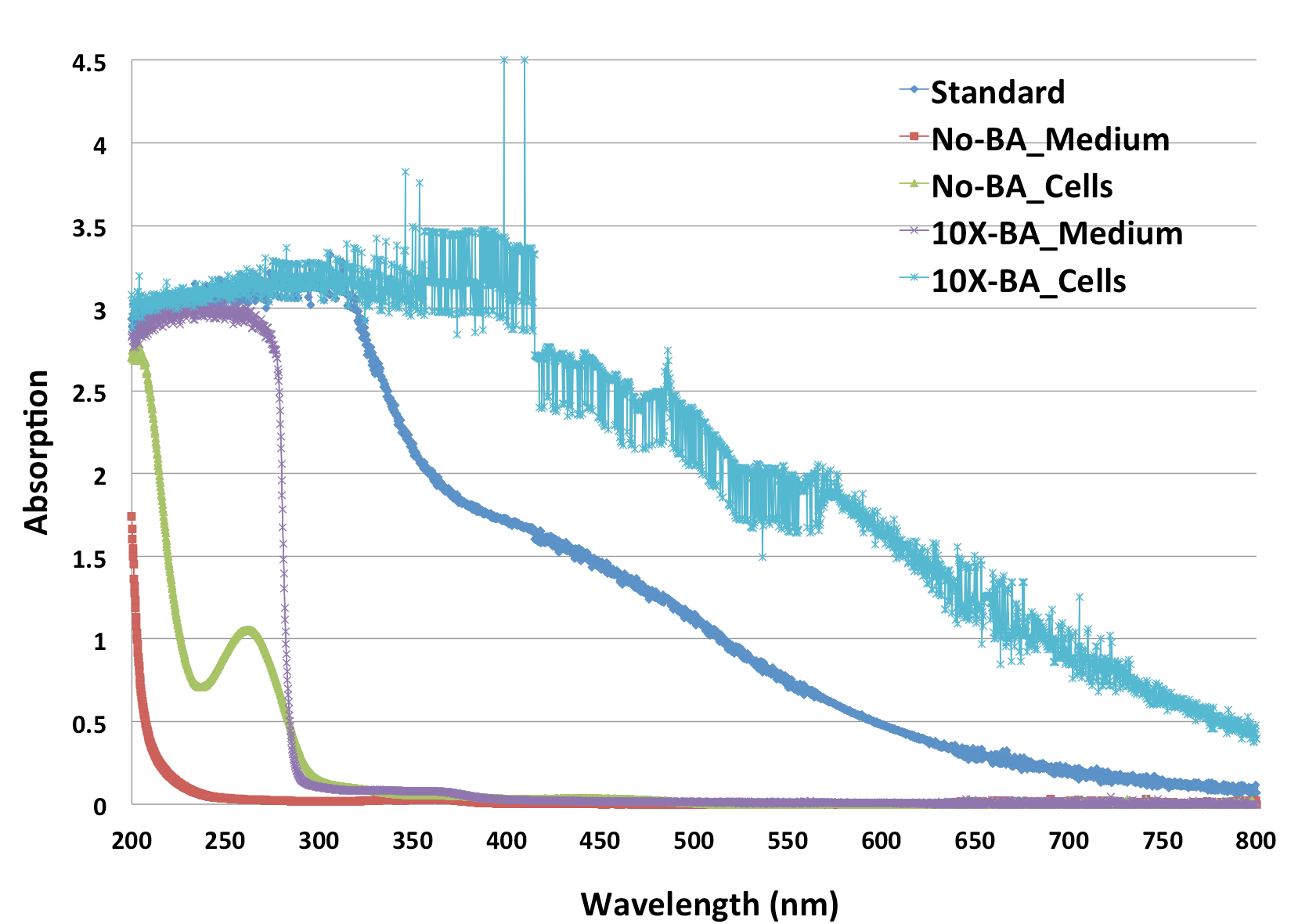

Supplement: Supplementary file 1 [file DataSheet1.ZIP › 80907_Wang_Presentation_1/80907_Wang_Figure_S1.TIFF]
